# Adolescent Maturational Transitions in the Prefrontal Cortex and Dopamine Signaling as a Risk Factor for the Development of Obesity and High Fat/High Sugar Diet Induced Cognitive Deficits

**DOI:** 10.3389/fnbeh.2016.00189

**Published:** 2016-10-13

**Authors:** Amy C. Reichelt

**Affiliations:** School of Health and Biomedical Sciences, RMIT UniversityMelbourne, VIC, Australia

**Keywords:** dopamine, adolescence, obesity, high fat diet, sucrose, prefrontal cortex, hippocampus, striatum

## Abstract

Adolescence poses as both a transitional period in neurodevelopment and lifestyle practices. In particular, the developmental trajectory of the prefrontal cortex (PFC), a critical region for behavioral control and self-regulation, is enduring, not reaching functional maturity until the early 20 s in humans. Furthermore, the neurotransmitter dopamine is particularly abundant during adolescence, tuning the brain to rapidly learn about rewards and regulating aspects of neuroplasticity. Thus, adolescence is proposed to represent a period of vulnerability towards reward-driven behaviors such as the consumption of palatable high fat and high sugar diets. This is reflected in the increasing prevalence of obesity in children and adolescents as they are the greatest consumers of “junk foods”. Excessive consumption of diets laden in saturated fat and refined sugars not only leads to weight gain and the development of obesity, but experimental studies with rodents indicate they evoke cognitive deficits in learning and memory process by disrupting neuroplasticity and altering reward processing neurocircuitry. Consumption of these high fat and high sugar diets have been reported to have a particularly pronounced impact on cognition when consumed during adolescence, demonstrating a susceptibility of the adolescent brain to enduring cognitive deficits. The adolescent brain, with heightened reward sensitivity and diminished behavioral control compared to the mature adult brain, appears to be a risk for aberrant eating behaviors that may underpin the development of obesity. This review explores the neurodevelopmental changes in the PFC and mesocortical dopamine signaling that occur during adolescence, and how these potentially underpin the overconsumption of palatable food and development of obesogenic diet-induced cognitive deficits.

## Introduction

### Adolescence, Fast Food and Adverse Psychological Conditions

Adolescence represents a transitional developmental period between childhood and adulthood in the human (and animal) lifespan. In humans, the World Health Organisation (WHO) identifies adolescence as the period in human growth and development that occurs after childhood and before adulthood, approximately between ages 10–19 (WHO), although it is typically defined as the period from puberty to legal adulthood, encompassing the teenage years (13–19). Longitudinal neuroimaging studies have demonstrated that the brain, in particular the prefrontal cortex (PFC), continues to mature until ~24 years old (Giedd et al., 1999; Sowell et al., 2001; Casey et al., 2008b; Wahlstrom et al., 2010; Mills et al., 2014). Brain maturation processes during adolescence are influenced by sex steroids, which increase during puberty (Sisk and Foster, 2004). Sex hormones augment neuronal myelination (Martini and Melcangi, 1991) and modulate the development of neurocircuitry that subserves high-order cognition, reward and emotional processing (Dahl, 2001; Zhou et al., 2002; Peper et al., 2011). Neurodevelopmental changes and cortical reorganization are needed for the occurrence of adult behaviors, however the adolescent brain is highly susceptible to neural insults, which may disturb the natural course of brain maturation and key processes of brain development. Thus, adolescence is also the time when symptoms of a variety of mental illnesses often manifest, including mood disorders, eating disorders and schizophrenia (Spear, 2000; Sisk and Foster, 2004; Paus et al., 2008).

Excessive consumption of highly palatable sugar and fat laden foods, often in the form of “junk” or “fast” foods plays a central role in the development of obesity in humans (Malik et al., 2013). Of greatest concern is that the prevalence of obesity is increasing among children and adolescents. In the last 30 years obesity has more than doubled in children and quadrupled in adolescents, with now more than one third of children and adolescents in the developed world classified as overweight or obese (Ogden et al., 2014). Dramatic modifications in lifestyle patterns, such as making independent food choices, occur during adolescence (Nielsen et al., 2002; Story and French, 2004). Fast foods are laden with refined sugars and saturated fats and due to their convenience and low cost are readily accessible to young people (Davis and Carpenter, 2009). Adolescents and young adults consume more fast foods in comparison to older adults (Nielsen et al., 2002). Reports indicated that North American college students aged 18–24 ate at fast food restaurants 1–3 times weekly (Morse and Driskell, 2009) and 75% of school age children consumed fast foods once a week (French et al., 2002). Furthermore, dietary intake of refined sugar is greatest in adolescents than any other age group (Bremer and Lustig, 2012).

Epidemiological studies have identified associations between obesity in young people and psychological conditions including impulsivity, anxiety, drug abuse and attention deficit hyperactive disorder (ADHD; Waring and Lapane, 2008; Pagoto et al., 2009; Cortese and Vincenzi, 2012). ADHD and obesity are proposed to share a similar underlying neurobiological dysfunction of the dopaminergic system. In particular, a high incidence of comorbidity between ADHD and obesity has been observed (Altfas, 2002; Cortese et al., 2008). The behavioral manifestation of impulsivity in ADHD potentially contributes to weight gain via dysregulation of eating patterns (Erhart et al., 2012). A significant link between ADHD in adolescents and excessive consumption of “Western” junk food diets has been observed, indicating that adolescents with ADHD symptoms had a significantly higher intake of dietary fat, sugar and sodium than a traditional healthy diet (Howard et al., 2011). The co-occurrence of ADHD and obesity may be due to a common genetic and neurobiological pathway. Reports indicate that ADHD coincided with the expression of certain obesity-related genes in the pathways of dopaminergic neurocircuitry, such as fat mass–and obesity-associated variant (FTO; Albayrak et al., 2013) and melanocortin-4 receptor (MC4R; Agranat-Meged et al., 2008). Polymorphisms of these genes have been linked with the incidence of obesity and dysregulated eating behaviors in humans (Frayling et al., 2007; Gerken et al., 2007; Cecil et al., 2008; Peng et al., 2011; Yilmaz et al., 2015). In particular the MC4R rs17782313 polymorphism is implicated in emotional eating and food cravings (Yilmaz et al., 2015) and the FTO rs1558902 polymorphism is associated with binge eating in adolescence (Micali et al., 2015).

### Palatable Food as a Rewarding Substance

Consumption of palatable high fat and high sugar foods leads to activation of the brain’s reward neurocircuitry, the mesocorticolimbic dopamine system (Del Parigi et al., 2003; Avena et al., 2006, 2008; Kenny, 2011), resulting in the extracellular release of dopamine in regions including the nucleus accumbens and PFC. These regions receive long axon dopamine projections originating from the ventral tegmental area that form the mesocorticolimbic reward system. Dopamine acts within and across limbic, striatal and frontal neurocircuitry to promote and regulate motivated behavior, especially food seeking (Depue and Collins, 1999). Excessive consumption of sugar and fat rich foods evokes enduring changes in dopamine signaling within regions involved in reward processing, cognitive functions and motivation, including the nucleus accumbens (Rada et al., 2005; Sharma et al., 2013), the hippocampus (Kaczmarczyk et al., 2013; Krishna et al., 2015) and the PFC (Wakabayashi et al., 2015).

### Maturation of the Dopaminergic Reward System During Adolescence

Adolescence has been described as a period of heightened affective reactivity characterized by an increased sensitivity to natural rewards (Van Leijenhorst et al., 2008, 2010; Somerville et al., 2010; Crone and Dahl, 2012), including palatable foods (Spear, 2000; Wilmouth and Spear, 2009; Friemel et al., 2010). Age-dependent changes in fronto-striatal cortical maturation may underlie the increased sensitivity to palatable food rewards during adolescence (Friemel et al., 2010; Crone and Dahl, 2012). The rapid growth spurt that occurs in puberty and adolescence provides partial protection against diet-induced obesity, but a large tendency for hyperphagia (Spear, 2000; Labouesse et al., 2013). As such, chronic stimulation of the still maturing mesocorticolimbic dopamine system during adolescence by excessive consumption of palatable foods is hypothesized in this manuscript to increase vulnerability to psychiatric disorders (Zametkin et al., 2004), pathological eating behaviors (Smith and Robbins, 2013) and cognitive dysregulation (Liang et al., 2014).

Studies using rodents and non-human primates provide neurochemical, structural and electrophysiological evidence signifying that the reward-signaling dopaminergic innervation originating from the ventral tegmental area to the PFC and nucleus accumbens matures during adolescence (Tseng and O’Donnell, 2007b; Benoit-Marand and O’Donnell, 2008; Brenhouse et al., 2008; Wahlstrom et al., 2010; Mastwal et al., 2014; Palm and Nylander, 2014). These observations have significant implications for adolescent-onset drug addiction (Palmer et al., 2009), but also may elucidate why reward driven behaviors such as excessive overeating leading to obesity is increasingly abundant in young people (Lee and Gibbs, 2013).

#### Dopamine Release During Adolescence

The dopaminergic innervation of the PFC peaks during adolescence in rats and monkeys (Kalsbeek et al., 1988; Rosenberg and Lewis, 1994, 1995; Tarazi and Baldessarini, 2000) and neurochemical changes evoked by psychostimulants are distinct during this stage (Andersen et al., 2001; Tirelli et al., 2003). Primate and rodent studies have indicated increased levels of functionally available dopamine during adolescence, though differences exist with respect to the neuroanatomical regions and aspects of the dopamine system affected between species. Increased cortical and subcortical tissue concentrations of dopamine are observed during juvenile and adolescent periods in monkeys (Goldman-Rakic and Brown, 1982; Irwin et al., 1994; Caballero et al., 2016). In rodents, including mice and rats, adolescence, comprising the pubertal period, extends across postnatal days (P) 28–42, however functional alterations occur into late adolescence (P56) including maturation of the rodent homolog of the primate PFC and changes in frontostriatal dopamine signaling (Spear, 2000; Caballero et al., 2016; Hunt et al., 2016). Dopamine levels in the rodent brain increase in the striatum during adolescence (Teicher et al., 1993; Andersen et al., 1997), this is proposed to result from a reduced basal rate of dopamine release in adolescents relative to adults (Stamford, 1989; Andersen and Gazzara, 1993). However, when stimulated by environmental or pharmacological challenges, dopaminergic neurons in the adolescent brain release more dopamine than adults measured by microdialysis (Laviola et al., 2001). This indicates that during adolescence rewarding events such as consuming palatable foods may result in larger dopamine release in comparison to adulthood (Laviola et al., 2003).

#### Dopamine Receptors During Adolescence

**Figure 1:**
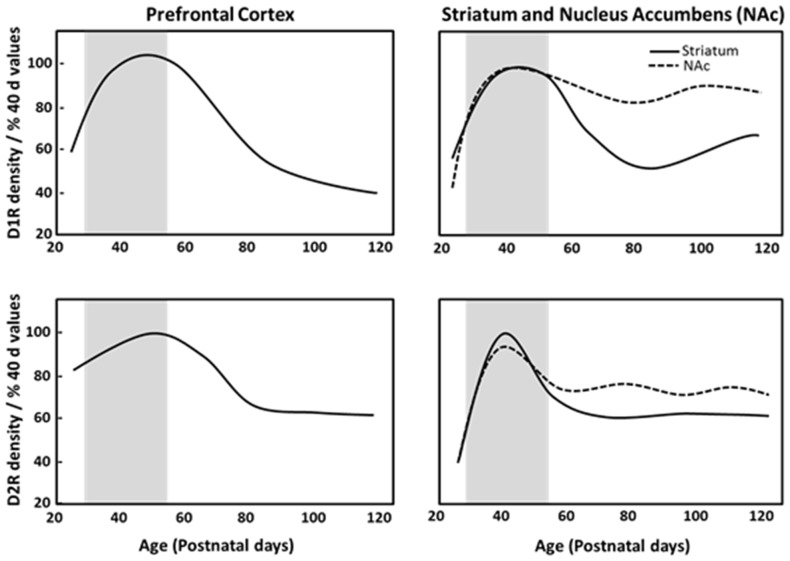
**Density of dopamine D1 (D1R) and D2 receptors (D2R) in the prefrontal cortex (PFC), striatum and nucleus accumbens of male Sprague Dawley rats at specific postnatal days (P), based on Andersen et al. (2000).** Gray region depicts adolescence as P28–56.

Dopamine receptors are overproduced and then pruned during adolescence in fronto-striatal regions (Teicher et al., 1995). Postmortem analysis of human brain tissue has reported developmental declines in dopamine receptor populations in striatal regions during adolescence, with approximately one-third to one-half of the dopamine receptor 1 (D1R) and dopamine receptor 2 (D2R) present in the striatum of children being lost by adulthood (Seeman et al., 1987; Palacios et al., 1988). Similarly, in monkeys, cortical and subcortical D1R and D2R density peaks in childhood, and decreases across adolescence into adulthood (Seeman et al., 1987; Lidow and Rakic, 1992). Rodent anatomical studies demonstrated that D1R and D2R density peaks in adolescence and then declines across adulthood in the striatum and PFC (Teicher et al., 1995; Andersen et al., 2000; Tarazi and Baldessarini, 2000; Brenhouse et al., 2008). Autoradiography studies have indicated that pruning of about one-third of D1R and D2R in the dorsal striatum and nucleus accumbens during adolescence (Teicher et al., 1995; Tarazi et al., 1998, 1999), and PFC D1R density and associated second messenger activity rises dramatically between P25 (juvenile) and P40 (adolescence), with a subsequent reduction by P100 (full adulthood) in rats as shown in Figure 1 (Teicher et al., 1995; Andersen et al., 2000). Anatomical studies have shown that dopaminergic innervation of the PFC increases progressively until P50–60 (Verney et al., 1982; Kalsbeek et al., 1988; Benes et al., 2000) and D2R/D4R expression reaches a stable adult level at P35 (Tarazi et al., 1998; Tarazi and Baldessarini, 2000). This pinpoints adolescence as a period of substantial change in the dopaminergic reward pathways and cortico-accumbal neural connectivity. The heightened expression of D1R on cortico-accumbal projections further supports increased sensitivity to environmental events and addictive behaviors during adolescence (Laviola et al., 2003; Brenhouse et al., 2008).

#### Development of Inhibitory Neurotransmission Across Adolescence

D1R stimulation is a major modulator of synaptic plasticity in the PFC, hence may regulate synaptic connectivity (Huang et al., 2004). During adolescence, dopamine is critical in controlling balance between excitatory and inhibitory neurotransmission in the PFC (Tseng et al., 2007;Tseng and O’Donnell, 2007a). In the postnatal mammalian brain, γ-aminobutyric acid (GABA) is the principle inhibitory neurotransmitter and glutamate is the principle excitatory neurotransmitter. Excitatory and inhibitory neurotransmission balance in the mature PFC is critical in cognition and the control of behavior (Yizhar et al., 2011; Lewis et al., 2012; Nelson and Valakh, 2015). Neurochemical evidence indicates that GABAergic neurotransmission, particularly within the PFC, remains under construction during adolescence (Lewis, 1997; Crews et al., 2007; Tseng and O’Donnell, 2007a; Caballero and Tseng, 2016). This delayed development of GABAergic neurotransmission may underpin behaviors such as increased risk-taking in adolescence (Van Leijenhorst et al., 2010; Schindler et al., 2016) and susceptibility to development of psychiatric disorders during this period (Caballero and Tseng, 2016).

The dopaminergic innervation of PFC glutamatergic pyramidal neurons and GABAergic interneurons and their interaction matures during adolescence (Tseng and O’Donnell, 2007a) and controls aspects of reward processing. Parvalbumin expressing neurons are a major class of GABAergic interneurons. A recent study indicated reduced parvalbumin immunoreactivity in the PFC and dorsal hippocampus of rats that consumed a high sucrose diet for 28 days across adolescence (Reichelt et al., 2015). This suggests that high sucrose diets may reduce GABAergic inhibition in these brain regions, potentially underpinning diet-evoked cognitive deficits manifesting as dysregulation of behavioral control (Reichelt et al., 2015), however further studies are needed to establish whether these effects are specific to adolescent diet-exposure. Sonntag et al. (2014) utilized virally mediated elevation of PFC D1R on glutamatergic neurons in adult rats (CamKII.D1) to recapitulate the increased PFC D1R levels in adolescence (see Figure 1, Andersen et al., 2000). The lentiviral induced elevation of D1R functionally resulted in greater consumption of ascending concentrations of sucrose (0, 0.25, 0.5 and 1%) or 0.1% saccharin using a two-bottle choice task between the solutions and water, indicative of increased sensitivity to the rewarding properties of sweet liquids in CamKII.D1 rats (Sonntag et al., 2014). Furthermore, a delayed discounting task was conducted in a T-maze where the animals had the choice between an immediate small reward or waiting across a delay period for a large reward (1 or 4 pieces of Reece’s Pieces respectively), increased choices for the small reward rather than waiting for the larger reward was observed in the CamKII.D1 rats, indicating increased impulsivity (Sonntag et al., 2014). Elevation of cortical D1R also resulted in decreased D2R expression in the nucleus accumbens (Sonntag et al., 2014), which is known to be a risk marker for obesity (Wang et al., 2004). The up-regulation of D1Rs in the PFC may therefore render adolescents selectively vulnerable to overeating compared with a normal adult population, and provides a potential mechanism for why reduced D2Rs are observed in the nucleus accumbens of obese subjects.

#### Development of other PFC Reward-Processing Neurotransmitter Systems During Adolescence

Multiple neurotransmitter systems are also developing during adolescence, particularly those projecting to the PFC. This includes the acetylcholinergic system that is critical for reward processing and cognitive processes. Neuronal nicotinic acetylcholine receptors (nAChRs) nAChRs exhibit distinct patterns of expression that parallel key developmental events within the cholinergic system and are critical regulators of brain maturation from prenatal development through adolescence (Dwyer et al., 2009). In rodents, expression and binding at α4β2 and α7 nAChR subtypes is higher in many brain regions in adolescents than in adults (Adriani et al., 2003; Doura et al., 2008). Neuronal nAChRs centrally regulate signaling in reward pathways (Dani and Balfour, 2011); and nAChR activation modulates dopamine release, which is strongly implicated in reward processing and reinforcement (Gotti et al., 2006; Albuquerque et al., 2009). Thus, in the adolescent brain, reduced regulation of dopamine by nAChRs may exacerbate reward-seeking, including palatable food consumption.

Furthermore, the endocannabinoid system has recently emerged as a regulator of PFC plasticity and undergoes age-dependent changes that directly impact PFC activity. CB1 receptor expression increases during postnatal development, with levels peaking at mid-adolescence (Schneider et al., 2008; Klugmann et al., 2011), which is accompanied by relative changes in the concentrations of endocannabinoids (Berrendero et al., 1998). Recent studies in rats indicate that repeated cannabinoid agonist administration during adolescence evokes neuronal deficits are at the level of PFC GABAergic circuitry, however this does not occur when cannabinoid agonist treatment occurs in adulthood, indicating that stimulation during adolescence hinders the appropriate development of the PFC (Cass et al., 2014). Notably, alterations to CB1 receptor expression has been observed in dietary obese rats (Bello et al., 2012), mice (South and Huang, 2008) and humans (Bordicchia et al., 2010). However, the exact mechanisms by which CB1 receptor signaling enables PFC maturation remain to be determined, as is whether adolescent diet induced alterations in CB1 receptors alter PFC maturation.

#### Summary—Neurodevelopmental Impact of Reward System Stimulation by Palatable Foods

Following prolonged consumption of high fat and/or high sugar diets neuroadaptive effects have been observed in reward processing regions. Obesity is associated with deficits in dopamine neurotransmission, which may drive the overconsumption of palatable foods (Geiger et al., 2009). This manifests as down-regulation of striatal D2R density in obese adult rats (Johnson and Kenny, 2010), and humans (Wang et al., 2001; Stice et al., 2008). Stimulation of the mesocorticolimbic dopaminergic system during adolescence by the consumption of highly palatable foods may potentially impact on cortico-striatal maturation, altering the age-associated pruning of frontostriatal D1R/D2R (Andersen et al., 2000), dysregulating PFC function and altering reward-processing. However, currently no studies have directly compared D1R and D2R densities in the forebrain between adolescent and adult animals exposed to high fat and/or high sugar diets. From drug addiction literature it is proposed that reduced top-down dopaminergic innervation from the PFC to the nucleus accumbens and striatum may increase the motivation to procure rewards (Volkow et al., 2012). In drug addiction, the enhanced incentive value of the drug in the reward, motivation, and memory circuits overcomes the inhibitory control exerted by the PFC, promoting consumption of the drug due to enhanced activation of motivational neurocircuitry (Volkow et al., 2012). Therefore, in the setting of reward-driven eating behavior, the reduced inhibitory control from the PFC may permit the overconsumption of palatable foods observed in adolescents (Tomasi and Volkow, 2013; Volkow et al., 2013).

## Behavioral and Cognitive Changes Following Adolescent High Fat/High Sugar Diet Consumption

Excessive consumption of palatable high fat/high sugar foods may exacerbate cognitive deficits including impulsivity and impaired decision-making (Crews et al., 2007), evoke enduring reward processing alterations that continue into adulthood, and promote the development of addiction-like behaviors and obesity (Volkow et al., 2008; Johnson and Kenny, 2010; de Weijer et al., 2011; Tuominen et al., 2015). The following sections discuss experimental studies that have functionally examined the resultant behavioral and cognitive changes that are evoked by adolescent palatable high fat and/or high sugar diet consumption, and experiments that pinpoint adolescence as a period of diminished behavioral control. These studies support the hypothesis that adolescence is a period of vulnerability to diet induced cognitive and behavioral alterations.

### Impact of Adolescent High Fat and/or High Sugar Diets on Learning and Memory Processes

A growing body of experimental literature has indicated that adolescence may be a critical period for the development of pronounced and enduring cognitive and behavioral alterations following exposure to alcohol (Nasrallah et al., 2009; Gass et al., 2014; Schindler et al., 2014), psychostimulants (Sherrill et al., 2013; Hammerslag et al., 2014) cannabinoids (Schneider et al., 2008) and high fat or high sugar diet consumption (Boitard et al., 2012, 2014, 2015; Reichelt et al., 2015). Exacerbated cognitive deficits in young rats and mice are reported following consumption of diets rich in saturated fats and/or refined sugars commencing in early life, as juveniles or adolescents, when compared to animals that commenced exposure to the same diets during adulthood.

Studies have characterized diet-induced learning deficits in hippocampal-dependent long term memory formation (assessed in the radial-arm maze or Morris water maze) when high fat diet consumption began during the juvenile/adolescent period in both rats (Greenwood and Winocur, 2005) and mice (Valladolid-Acebes et al., 2011). This memory impairment was not observed when diet access started in 8 week old rodents (P56; Mielke et al., 2006; White et al., 2009; McNeilly et al., 2011), which is considered to be the end of adolescence in rodents.

Studies have reported consumption of high fat and/or high sugar diets leads to cognitive deficits in PFC mediated behaviors, particularly those requiring modulation of attention towards stimuli. McNeilly et al. (2011) measured working memory in animals fed a high fat diet in an operant delayed match to position test shown to be sensitive to PFC function (Sloan et al., 2006). In this task animals had to respond to one lever “sample” then after a 5 s delay, a choice phase of the presentation of two levers which was correct if the original lever was depressed “match to position”. High fat diet fed rats were impaired at this task, however these deficits were further exacerbated when the task was switched to a delayed non-match to position task, whereby the correct lever was the different lever to the sample “non-match to position”. This indicated a deficit in behavioral flexibility in high fat diet fed rats, in particular, reversing a learned contingency.

Prepulse inhibition (PPI), which is the ability to filter out sensory information and is subserved by the PFC and striatum, can be measured by an acoustic startle response. Typically, the reduction of the startle response amplitude to an intense stimulus is observed if there by a preceding weak, non-startling stimulus. This blunted startle response was shown to be impaired in adult mice fed a high fat diet (Labouesse et al., 2013; Wakabayashi et al., 2015) and these deficits in PPI were linked to decreased PFC D1R in high fat diet fed mice (Wakabayashi et al., 2015). Furthermore, the ability to reduce, or block, learning about a stimulus when it is paired in compound with another stimulus already associated with an outcome depends on the PFC (Fletcher et al., 2001) and midbrain dopamine signaling (Waelti et al., 2001). An appetitive blocking task was conducted in adult rats that had consumed sucrose in a binge-like manner for 28 days (Sharpe et al., 2016), the sucrose exposed animals, but not controls, approached a cue signaling food delivery that is usually blocked by prior learning, an effect dependent on dopaminergic prediction-error signaling in the midbrain. It appeared that disruption of PFC dopaminergic signaling following consumption of sucrose supplemented diets contributed to the behavioral alterations observed. In particular, intraventricular infusion of the D2R agonist quinpirole restored PFC-mediated cognitive control of learning about food cues in adult rats with a history of sucrose binging. This provides evidence of diet-induced alterations to D2R signaling mechanisms in the PFC, potentially due to a down-regulation of D2R (Sharpe et al., 2016).

Functional connectivity between the hippocampus and PFC is essential for many executive cognitive functions (Floresco et al., 1997; Thierry et al., 2000). Disruption of hippocampal–PFC synchrony is associated with the cognitive deficits that occur in neuropsychiatric disorders such as schizophrenia (Dickerson et al., 2010, 2012; Sigurdsson et al., 2010), and D2R-dependent control of glutamatergic NMDAR neurotransmission has been particularly implicated in the regulation of hippocampal-PFC functional connectivity (Banks et al., 2015). More so, presynaptic D2Rs in the hippocampus modulate long-term depression and aspects of long-term potentiation expression and functionally regulate hippocampally dependent learning and memory performance (Rocchetti et al., 2015). Thus, alterations to D2R signaling by excessive consumption of high fat and/or high sugar diets may underpin aspects of diet-induced cognitive deficits.

Highly controlled studies using animal models have revealed that consumption of high fat diets during the juvenile and adolescent period of life negatively impact memory function to a greater degree than adult consumption of the same diets. Rats exposed to a high fat diet (45% kcal from fat) from weaning (i.e., 3 weeks of age) as juveniles, but not adult rats, were observed to be impaired at spatial memory retention and spatial reversal learning (Boitard et al., 2014). Similarly, mice fed a high fat diet for 11 weeks post-weaning were impaired at memory flexibility assessed in a two-stage radial arm maze concurrent spatial discrimination task, whereas adults exposed to the same high fat diet beginning at 12 weeks old (P84) and for the same duration of diet exposure did not show cognitive deficits on this task (Boitard et al., 2012). Adolescent mice aged 5 weeks old at commencement of diet access were impaired in the hippocampal dependent novel location recognition task and these cognitive deficits continued after 5 weeks of diet restriction, whereas mice that were given the diet at 8 weeks old were not impaired (Valladolid-Acebes et al., 2013). Similarly, rats that consumed sugar supplemented diets in the form of either 11% sucrose solution or 11% high fructose corn syrup (HFCS) solution for 30 days across adolescence had hippocampal dependent learning impairments, which were not observed in adult rats consuming the same high sugar diets (Hsu et al., 2015). Protein expression of the pro-inflammatory cytokines IL-1β and IL-6 were increased in the hippocampus of adolescent HFCS consuming rats, but this was not observed in adult rats (Hsu et al., 2015). This indicates a particular vulnerability of the adolescent hippocampus to neuroinflammation following consumption of high sugar diets.

Adolescence is also considered a period of enhanced emotional reactivity to stressful and arousing events. Long term emotional memories are underpinned by limbic regions including the basolateral amygdala (BLA; McGaugh, 2004), and influenced by function of the hypothalamic–pituitary–adrenal (HPA) axis (Roozendaal et al., 2006). Obesity induced alterations to the HPA axis is observed in humans (Pasquali et al., 2006) and rodent models (Sharma et al., 2013), leading to an exaggeration of emotional responses. In rats and mice, emotional responses can be measured by fear conditioning preparations, whereby a stimulus such as a tone is presented prior to a mild footshock. Following conditioning, animals will elicit fear responses in the form of freezing when the tone is presented, indicative of learning an association between the stimulus and the aversive event. Furthermore, emotional responses can also be assessed by pairing the consumption of an odorized water solution with lithium chloride induced malaise. Animals will then avoid consuming the odorized solution as they associate it with unpleasant malaise. Emotional memories were examined in rats that consumed a high fat diet across adolescence, but not in adulthood. It was observed that a high fat diet consumed across adolescence enhanced the expression of emotional memories assessed by odor-malaise, the rats that consumed high fat diets as adolescents formed a stronger aversion to the malaise-paired solution in comparison to adult rats that consumed high fat diets, and age-matched control animals (Boitard et al., 2015). Furthermore, freezing to the shock paired stimulus was increased in adolescent rats that consumed a high fat diet, whereas rats that consumed the high fat diet as adults showed similar levels of freezing to control diet rats (Boitard et al., 2015). This demonstrated exacerbated emotional memory learning in high fat diet consuming adolescent, but not adult rats (Boitard et al., 2015). The PFC regulates fear behavior by modulating the activity of the amygdala (Paré et al., 2004; Chan et al., 2011), therefore the enhanced emotional memories observed in adolescent may be underpinned by reduced regulation of the BLA by the immature adolescent PFC. This indicated a potential vulnerability of adolescence to the development of increased emotional learning that may lead to the development of anxiety disorders.

#### Reduction of Hippocampal Neurogenesis Following Adolescent High Fat/High Sugar Diet Consumption

Hippocampal neurogenesis, and more specifically the integration of adult-born neurons into the hippocampal circuitry, is important in learning and memory processes (Koehl and Abrous, 2011) and is shown to be reduced in high sugar (Van der Borght et al., 2011; Reichelt et al., 2016b) and high fat diet consuming rodents (Park et al., 2010; Boitard et al., 2012). Neurogenesis occurs at higher levels in the hippocampus during adolescence compared to adulthood (Crews et al., 2007). Chronic alcohol consumption during adolescence induced long-term changes persisting into adulthood such as reduced neurogenesis marker expression in the dentate gyrus and depressive-like behaviors (Briones and Woods, 2013), and impaired object recognition memory (Vetreno and Crews, 2015). Similarly, mice that consumed a high fat diet across adolescence for 11 weeks had reduced levels of hippocampal neurogenesis measured by doublecortin immunoreactivity in the dentate gyrus compared to controls, but doublecortin immunoreactivity did not differ between high fat diet consuming adult mice and age-matched controls (Boitard et al., 2012).

In summary, the adolescent brain appears particularly vulnerable to the neurobiological impact of high fat and/or high sugar diet consumption. Collectively these studies strongly suggest that neural substrates of learning and memory, particularly the hippocampus, in the adolescent brain are susceptible to persistent neurobiological changes caused by overconsumption of palatable high fat and/or high sugar diets, and this may be due to an enduring reduction in neurogenesis, or increased neuroinflammatory reactions.

### Impact of Adolescent High Fat/High Sugar Diets on Reward-Directed Behaviors

Developing dopaminergic neurotransmission, particularly in the PFC, is associated with reward-seeking behavior during adolescence (Wahlstrom et al., 2010). An important question is whether consumption of palatable foods impacts the developing adolescent brain in a different way and with longer-lasting consequences compared to the mature adult brain. The affective components of rewards such as palatable high fat/high sugar foods can be broken down into *wanting* and *liking* (Berridge and Robinson, 1998; Barbano and Cador, 2007; Castro and Berridge, 2014), whereby wanting refers to the motivational component of reward, and liking refers to the hedonic component of reward. Thus, the affective impact of rewarding substances during development can be examined separately by how motivated an animal is to procure the substance, that is, the incentive salience attributed the reward, and the hedonic appraisal of the substance. Neuroimaging studies in humans demonstrate greater reactivity within the nucleus accumbens in adolescents following food reward deliveries relative to young children and adults (Galvan et al., 2006; Geier et al., 2010), indicating a particular sensitivity to rewards in adolescents. This increased sensitivity to the rewarding properties of palatable foods and drinks observed in adolescents may promote hyperphagia (Bernheim et al., 2013), and due to this increased “dose” of rewarding foods consumed, alterations to neuronal processes may also be more pronounced. Under experimental conditions this hyperphagic behavior is supported by reports of enhanced consumption of palatable sucrose solutions by adolescent rats as a factor of body weight in comparison to adult animals (Kendig et al., 2013) and may be underpinned by an increased positive taste responsivity in adolescent rats (Wilmouth and Spear, 2009).

Motivation to procure an outcome typically depends on the strength, or reinforcing efficacy, of the reward, which can be measured by progressive ratio schedules (Richardson and Roberts, 1996). Under progressive ratio schedules the requirements (i.e number of lever presses) to earn a reinforcement delivery are increased systematically, usually after each reinforcer. It was demonstrated that male rats that had their diet supplemented with 5% sucrose solution continuously in their homecages during adolescence, but not in adulthood, were less motivated to perform lever press responses on a progressive ratio schedule for palatable food rewards when tested as adults (Vendruscolo et al., 2010), indicating sucrose consumption during adolescence evoked long term changes in reward processing. However, another recent study indicated that observation of reduced motivation measured by progressive ratio following adolescent sucrose consumption may be specific to male rats. (Reichelt et al., 2016a). In this study, female rats that consumed 10% sucrose for 2 h a day across adolescence showed increased motivation measured by an increased breakpoint of lever presses to procure palatable rewards when tested as adults, however male rats showed reduced breakpoints on a progressive ratio schedule (Reichelt et al., 2016a). It should also be noted that in both the studies by Vendruscolo et al. (2010) and Reichelt et al. (2016a), behavioral alterations were observed without differences in body weight between the experimental groups despite increased overall energy intake in sucrose consuming rats, thus behavioral changes cannot simply be attributed to altered motivation state due to different baselines of hunger during behavioral testing. Naneix et al. (2016) recently assessed the effect of sucrose consumption across adolescence in adult male rats on the hedonic impact of sweet rewards (sucrose and saccharin) by affective reactions, measured by orofacial reactions to intraoral infusions. This study indicated that daily sucrose intake during adolescence led to decreased positive orofacial responses to sweet tastes when the rats were assessed as adults (Naneix et al., 2016) and this hedonic deficit was associated with lower c-Fos expression levels in the nucleus accumbens. Thus, the hedonic appraisal of both caloric and non-caloric sweet rewards was lessened following adolescent sucrose consumption, suggesting a long-lasting lower hedonic state that may contribute to the development of reward-related disorders in adulthood (Naneix et al., 2016), which may potentiate the overconsumption of palatable foods (Johnson and Kenny, 2010). In a human setting, the hedonic response to palatable sweet tastes of ascending concentrations of sucrose solution measured by a self report questionnaire was associated with elevated sensitivity to the mood altering effects of sweet foods and impaired control over eating sweets, with a greater preference for concentrated sucrose observed in women (Kampov-Polevoy et al., 2006).

Conditioned place preference (CPP) studies pair one discriminable context with the administration of a rewarding substance, and another with no reward, so that the animals come to prefer the reward paired context paired when presented with a choice between the two environments. Rats and mice prefer and approach environmental cues that are associated with consumption of a palatable food reward (Perello et al., 2010). Through repeated pairings of access to palatable foods in a certain environment, animals will elicit an approach response to the food-rewarded environment in comparison to a control, non-food paired environment. Consumption of a high fat diet across adolescence (P21–40), but not as adults (P61–80), evoked long lasting impairments in CPP for a palatable food (Cheetos) in male rats (Privitera et al., 2011). This suggests that rats that consumed palatable food during adolescence learned less about the environment that the food reward was presented in. This may be indicative that the adolescent high fat diet consuming rats found the palatable food less rewarding, or that they showed deficits in encoding the environmental features associated with the food rewards.

These studies indicate that high fat or high sugar diets across adolescence evoked alterations in both motivation and hedonic appraisal of food rewards in adulthood, processes subserved by reward-processing regions including the striatum and nucleus accumbens. This may increase the risk of developing neuropsychiatric disorders, including depression, eating disorders and addiction, as well as obesity (Blundell and Finlayson, 2004; Marmorstein et al., 2014), which commonly emerge during adolescence (Pine et al., 1998; Paus et al., 2008).

### Adolescence as a Period of Impaired Behavioral Control

Instrumental behavior, such as performing a lever press action that is reinforced by a palatable outcome, is controlled by two discrete behavioral and neuronal systems: a stimulus-response habit mechanism and a goal-directed (action-outcome) process (Adams and Dickinson, 1981; Balleine and Dickinson, 1998; Balleine and O’Doherty, 2010). Habitual behaviors are generally inflexible, while the action-outcome is a dynamic process with a continuous and flexible feedback over performance of actions to acquire outcomes allowing behavior to adapt to changing environments (Adams and Dickinson, 1981; Balleine and Dickinson, 1998). In rats, the capacity to detect changes in action–outcome contingencies is governed by a neural circuit including the prelimbic PFC (Balleine and Dickinson, 1998) and the posterior dorsomedial striatum (pDMS; Yin et al., 2005). Furthermore, this capacity depends on dopamine signaling in the pDMS (Lex and Hauber, 2010a,b; Braun and Hauber, 2012). Performance of instrumental actions in rats is initially goal-directed and therefore sensitive to changes in reward value. However, after extended training stimulus-response habits emerge that are no longer goal-directed and are insensitive to changes in incentive value or action-outcome contingencies. Habitual responses are subserved by the infralimbic PFC (Balleine and Dickinson, 1998; Killcross and Coutureau, 2003) and dorsolateral striatum (DLS; Yin et al., 2004). Dopamine is known to play a role in the development of habits, whereby sensitization of the dopamine system in adult rats by chronic d-amphetamine treatment prior to instrumental training leads to an acceleration of habit formation (Nelson and Killcross, 2006), and dopamine depleting 6-OHDA lesions of the nigrostriatal dopamine system decreases habit formation (Robbins et al., 1990; Faure et al., 2005).

A large body of literature demonstrates that the adolescent period affords vulnerability to the higher-order control of behaviors. The following sections discuss experimental evidence demonstrating that adolescence is a distinctive period characterized by altered behavioral regulation of actions and stimuli associated with obtaining food rewards. The control of goal-directed instrumental actions is proposed to depend on the dopamine system (Balleine and O’Doherty, 2010). Evidence for contingency learning comes from demonstrations that instrumental performance is sensitive not only the probability of contiguous reward but also to the probability of unpaired rewards (Balleine and Dickinson, 1998; Braun and Hauber, 2012). Rats are initially trained to press on two separate levers for a reward that is delivered in a contingent manner—the action leads to the outcome. However, during a contingency degradation test, responding on one lever becomes degraded, as rewards are delivered randomly in a non-contingent manner to the action performed, and the rat should typically cease to respond to the lever, but maintain responding to the lever that responses are contingent to outcome deliveries. It was observed that adolescent rats failed to adapt their response to changes of action–outcome relationships, however these rats adapted to the contingency degradation protocol once adults, which paralleled the maturation of the cortical dopamine system (Naneix et al., 2012). This failure to update action-outcome contingencies in adolescent rats is potentially due to a diminished ability to encode the alteration in contingency due to immaturity of the PFC, as action-outcome contingencies has been shown to depend on dopamine signaling in the prelimbic PFC and DMS (Lex and Hauber, 2010b). The immaturity of the PFC and delayed development of the mesocortical dopamine pathway that projects to the PFC during adolescence is proposed to underpin sub-optimal decision making in the selection and execution of actions according to their predicted consequences (Naneix et al., 2012, 2013). Furthermore, treatment with the D2R agonist quinpirole across adolescence impacts on the developing dopamine system, decreasing dopamine fiber density, dopamine tissue concentration and dopamine receptors expression in the PFC (Naneix et al., 2013). The behavioral consequence of D2R stimulation was that adult rats treated with quinpirole showed behavioral deficits in updating actions during a contingency degradation test (Naneix et al., 2013), mimicking the deficits observed in adolescent rats (Naneix et al., 2012).

Poor behavioral control, lack of inhibition and impulsivity contribute to the propensity for adolescents to engage in risk-taking behaviors. Inhibiting a response can be assessed by a differential reinforcement of low-rate (DRL) schedule. This task requires animals to withhold a food-procuring response over a set period of time, before the response will be reinforced. Adolescent male rats were less sensitive to both the extinction of a learned response to obtain a palatable chocolate milk reward and to withhold a response on a DRL schedule indicating impaired behavioral inhibition in adolescent animals compared to adults. (Andrzejewski et al., 2011).

Reaction time tasks, such as the five-choice serial reaction time task (5-CSRTT) can be used to assess attention in rodents. Animals are required to attend a visual array that briefly presents a cue (illuminating a light across a 5 light array) to which responding is reinforced with a palatable reward, whilst inhibiting responses during the inter-trial period (Robbins, 2002). This task is shown to be dependent on dopaminergic neurotransmission within the medial PFC (Burton and Fletcher, 2012). Adolescent rats were trained to obtain a food pellet reward on a two-choice serial reaction time task (2-CSRTT), adolescents performed more impulsive actions in the form of premature responses in comparison to adults (Burton and Fletcher, 2012).

Studies examining choice behavior for large and small magnitudes of food rewards are used to recapitulate aspects of human gambling tasks, such as probability discounting tasks. These have noted a preference for large rewards in adolescent rats despite reductions in the probability in the delivery of the large reward (Zoratto et al., 2013). Choice performance can also be studied by requiring an animal to wait for a large reward over varying delays, or choose a small, immediate reward. Delay discounting describes the decrease in preference for a reward as a function of the delay to receiving it, so recapitulating elements of delayed gratification. Adolescent rats display more delay discounting, as they switch to the smaller reward associated response more rapidly than adult rats, indicative of increased impulsivity and inability to tolerate delays during adolescence (Doremus-Fitzwater et al., 2012).

A recent study examined the impact of high sugar diets on decision making in accordance to the presentation of discriminable stimuli that direct behavior. Consumption of 10% sucrose during adolescence reduced contextually appropriate responding to stimulus compounds in a biconditional discrimination task requiring the use of context as a task-setting cue (Reichelt et al., 2015). Rats first acquired two instrumental conditional discrimination in distinct contexts, one auditory (i.e A1→ R1, A2 → R2) in context 1 (C1; grid floor), and one visual (i.e V1→ R1, V2 → R2) in a different context (C2; bar floor). At test rats received compound stimuli that either comprised the auditory and visual elements that signaled the same lever response (congruent—A1V1, A2V2) or signaled different lever responses (incongruent—A1V2, A2V1) during training. During conflict (incongruent) trials, correct lever selection by control animals followed the stimulus element that had previously been trained in that same test context, whereas animals that had previously consumed sucrose failed to disambiguate the conflicting response cues. This task is sensitive to mPFC dysfunction (Haddon and Killcross, 2006; Reichelt et al., 2013b) and changes to dopaminergic signaling (Haddon and Killcross, 2011; Reichelt et al., 2013a), indicating a functional impact of sucrose-binging on decision making tasks.

Goal-directed behavioral control of instrumental responding can be assessed by conducting outcome devaluation by specific-satiety. This procedure provides experimental animals with the food outcome, or chow as a control, freely prior to the test session. During testing, animals are presented with the levers in extinction, that is, responses do not result in reward deliveries. It was observed that rats with a history of consuming a palatable food intermittently showed insensitivity to the devaluation treatment, and continued to lever press to procure the devalued outcome, indicative of stimulus-response habit formation, whereas control animals reduced lever pressing demonstrating goal-directed responding as the outcome is no longer valued (Furlong et al., 2014). However, whether consumption of high sugar diets during adolescence in comparison to maturity has a more pronounced behavioral impact on goal-directed instrumental responding has not been observed, as both sucrose-exposed adolescent rats and control adolescent rats showed reduced sensitivity to outcome devaluation by specific-satiety procedures (Kendig et al., 2013). This contrasts an earlier study demonstrating that adolescent rats were able to adapt their actions to changes in reward value following devaluation by sensory-specific satiety, but showed impairments in contingency degradation (Naneix et al., 2012).

As such, experimental studies with rats indicate adolescents are less able to regulate behavioral control over food reinforced actions, and in a human setting, this may predispose a tendency to consume palatable foods when posed with negative consequences such as weight gain and obesity, and intolerance to delayed gratification. Human studies have further identified that obese participants demonstrate risky patterns of decision-making on the Iowa Gambling Task (Brogan et al., 2010) and exhibited more impulsive patterns of choice for monetary outcomes than non-obese participants on delay discounting tasks (Lawyer et al., 2015).

#### Increased Stimulus-Directed Behavior in Adolescence

It has been suggested that adolescents may engage in more reward-seeking behaviors because their responses are biased towards stimulus-driven processes as opposed to the incentive value of an outcome (Ernst et al., 2011). This suggests that external, environmental cues or stimuli are more likely to direct attention and as such evoke a response (i.e., eating) which is independent of internal state (i.e., satiety/hunger; Ernst et al., 2011). In the laboratory setting, the accelerated development of stimulus-directed habitual behavior in adolescent rats has been observed (Hammerslag and Gulley, 2014). In this study, the interaction between age and sex on the expression of stimulus-directed behavior was assessed in rats using a Pavlovian conditioned approach paradigm. An auditory conditioned stimulus (CS+) was paired with delivery of a sucrose solution (unconditioned stimulus; US) to a food trough. The conditioned response (CR) to presentation of the CS+ measured by food trough entries was assessed during daily training sessions, following the devaluation of the reward by specific-satiety, and during periods of extinction and reacquisition. In this case, adolescent rats were less sensitive to outcome devaluation by specific-satiety, and exhibited a greater degree of reacquisition to an extinguished cue-reward association (Hammerslag and Gulley, 2014). The enhanced development of habitual responses to a food-associated stimulus is proposed to contribute to the vulnerability of adolescents to develop compulsive behaviors, such as binge eating (Hammerslag and Gulley, 2014).

In a human setting, adolescence has been noted as a period of particular reactivity to food-associated cues. Epidemiological studies have shown that adolescent obesity has tripled over the last three decades in the setting of food advertising directed at children (Flegal et al., 2016; Ogden et al., 2016). Studies have shown that the televised presentation of food-related advertisements increases food intake in children (Halford et al., 2004, 2007, 2008; Andreyeva et al., 2011; Kemps et al., 2014), and the increase in overweight and obese children has been linked to TV advertising of sugar and fat-dense junk foods (Lobstein and Dibb, 2005; Andreyeva et al., 2011; Lee et al., 2014). Advertising for food and beverages communicates food cues, priming the consumption of unhealthy foods and beverages (Harris et al., 2009; Lee et al., 2014). Adolescent youth appear especially sensitive to cue associated with rewards, as evidenced by exaggerated neural responses when exposed to them, specifically within structures innervated by mesolimbic dopamine (Galvan et al., 2006; Casey et al., 2008a; Hare et al., 2008; Bruce et al., 2010; Ernst et al., 2011). Obese adolescents displayed exaggerated neural responses measured by functional magnetic resonance imaging (fMRI) in striatal and limbic pathways upon exposure to high calorie food images vs. non-food images (Jastreboff et al., 2014). This observation suggested that obese adolescents show greater responsivity in reward related brain regions than lean adolescents to visual food stimuli, such as those commonly depicted in food advertising (Jastreboff et al., 2014).

#### Sucrose Craving in Adolescence

Sucrose is a highly rewarding substance, and sweet foods are often subject to cravings (Avena et al., 2008). Following prolonged withdrawal, or forced abstinence from a rewarding substance such as a drug of abuse, responding for that substance, or a cue associated with the substance, will increase when made available again, an effect referred to as “incubation of craving” (Lu et al., 2004; Pickens et al., 2011; Wolf, 2016). This effect has been observed in rats trained to lever press to receive deliveries of sucrose solution. The animals then undergo an “abstinence” period of days or weeks where they do not receive access to the training chambers where they learned to lever press for sucrose, which may or may not be preceded by extinction training where the sucrose is no longer delivered when a lever press response is made. Following the abstinence period, the rats typically reinstated lever press responding when returned to the training chambers, indicating that sucrose is subject to craving (Grimm et al., 2005, 2007; Uejima et al., 2007). Despite evidence of adolescent drug use resulting in an increased vulnerability to addiction (Palmer et al., 2009), studies in adolescent rats have indicated a diminished “craving”, measured by significantly lower rates of responding when returned to the instrumental chambers after the forced abstinence period compared to adult rats. This has been demonstrated after adolescent and adult rats were trained to self-administer cocaine following a 30-day abstinence period (Li and Frantz, 2009), heroin, following a 12-day abstinence period (Doherty et al., 2013) and under cue-induced sucrose-seeking conditions following a 21-day abstinence (Counotte et al., 2014). The subsequent attenuation of lever pressing elicited in adolescent rats contrasts the hypothesis that if adolescence affords an increased risk of addiction—craving should be greater, and the adolescent rats should perform more lever presses than adult rats following the enforced abstinence period. However, the reduced reinstatement of responding for sucrose solution adolescent rats is in-keeping with observations of diminished incubation of craving following self-administration of drugs of abuse (Li and Frantz, 2009; Doherty et al., 2013).

#### Summary—Adolescence as a Period of Impaired Behavioral Control

Experimental studies specify that adolescence is a period of enhanced responsiveness to rewards and reward-associated stimuli and responses. This is potentially due to increased risk-taking to gain rewards (Zoratto et al., 2013), the expression of behavioral responses that are insensitive to the contingency between actions and outcomes, and in some cases responses that are insensitive to the value of an outcome (Naneix et al., 2012; Hammerslag and Gulley, 2014). Furthermore, human neuroimaging studies have indicated increased neural responsivity to food-associated cues (Galvan et al., 2006; Casey et al., 2008a; Hare et al., 2008; Bruce et al., 2010; Ernst et al., 2011). These behaviors are underpinned by fronto-striatal regions and dopaminergic signaling mechanisms. Hence the impaired regulation of behavior observed in adolescents may potentiate the overconsumption of foods, driving the development of obesity.

## Conclusions and Implications

A range of animal and human experimental literature specifies that adolescence is a period of vulnerability to engage in rewarding, yet potentially risky, behaviors, including overconsumption of high fat and high sugar foods. This food preference and hyperphagia is partially underpinned by the still developing PFC and mesocorticolimbic dopamine system. The immature adolescent PFC impedes self-regulation during this life stage (Casey et al., 2008a; Blakemore and Robbins, 2012). More so, maturational changes occurring within the mesocorticolimbic dopamine system alters the sensitivity to the rewarding properties of palatable foods and drinks. Overconsumption of high fat and high sugar foods during adolescence may therefore impact the development of the PFC and mesocorticolimbic dopamine, leading to the pronounced behavioral alterations observed in tasks that rely on these systems when compared to adults consuming high fat and high sugar foods. Events that occur during sensitive periods such as adolescence may derail the normal maturation process and evoke a different trajectory of development, leading to an enduring predisposition towards certain behaviors (Andersen and Teicher, 2008; Paus et al., 2008). It is known that elevated PFC D1R play a significant role in increased motivational salience during adolescence (Brenhouse et al., 2008). In the setting of junk food diets, this may promote excessive consumption and increased reactivity to palatable food associated cues (Jastreboff et al., 2014), driving overconsumption during adolescence that extends into adulthood.

The physiological consequence of increasing global consumption of diets laden in fat and sugar are not simply the increasing prevalence of obesity, but also cognitive dysfunction, memory deficits and increased risk of developing psychiatric disorders in a younger population. Adolescence offers a period to identify key developmental processes that are amenable to intervention. This can create opportunities to identify and intervene in high-risk youth during periods where neural systems are more amenable to change, averting some of the destructive negative behavioral and cognitive spirals that may originate in adolescence. Thus, addressing the prevalence of high fat and high sugar diets in adolescents is vital, and further research should be undertaken to determine age-related cognitive effects of these diets and tailored intervention strategies.

## Author Contributions

ACR wrote and developed the manuscript.

## Conflict of Interest Statement

The author declares that the research was conducted in the absence of any commercial or financial relationships that could be construed as a potential conflict of interest. The reviewer WA and handling Editor declared their shared affiliation, and the handling Editor states that the process nevertheless met the standards of a fair and objective review.
